# Horizontal Movements, Migration Patterns, and Population Structure of Whale Sharks in the Gulf of Mexico and Northwestern Caribbean Sea

**DOI:** 10.1371/journal.pone.0071883

**Published:** 2013-08-21

**Authors:** Robert E. Hueter, John P. Tyminski, Rafael de la Parra

**Affiliations:** 1 Center for Shark Research, Mote Marine Laboratory, Sarasota, Florida, United States of America; 2 Proyecto Dominó – Ch’ooj Ajauil Asociación Civil, Cancún, México; University of Illinois at Chicago, United States of America

## Abstract

Whale sharks, *Rhincodon typus*, aggregate by the hundreds in a summer feeding area off the northeastern Yucatan Peninsula, Mexico, where the Gulf of Mexico meets the Caribbean Sea. The aggregation remains in the nutrient-rich waters off Isla Holbox, Isla Contoy and Isla Mujeres, Quintana Roo for several months in the summer and then dissipates between August and October. Little has been known about where these sharks come from or migrate to after they disperse. From 2003–2012, we used conventional visual tags, photo-identification, and satellite tags to characterize the basic population structure and large-scale horizontal movements of whale sharks that come to this feeding area off Mexico. The aggregation comprised sharks ranging 2.5–10.0 m in total length and included juveniles, subadults, and adults of both sexes, with a male-biased sex ratio (72%). Individual sharks remained in the area for an estimated mean duration of 24–33 days with maximum residency up to about 6 months as determined by photo-identification. After leaving the feeding area the sharks showed horizontal movements in multiple directions throughout the Gulf of Mexico basin, the northwestern Caribbean Sea, and the Straits of Florida. Returns of individual sharks to the Quintana Roo feeding area in subsequent years were common, with some animals returning for six consecutive years. One female shark with an estimated total length of 7.5 m moved at least 7,213 km in 150 days, traveling through the northern Caribbean Sea and across the equator to the South Atlantic Ocean where her satellite tag popped up near the Mid-Atlantic Ridge. We hypothesize this journey to the open waters of the Mid-Atlantic was for reproductive purposes but alternative explanations are considered. The broad movements of whale sharks across multiple political boundaries corroborates genetics data supporting gene flow between geographically distinct areas and underscores the need for management and conservation strategies for this species on a global scale.

## Introduction

The whale shark *Rhincodon typus* is the world’s largest living fish and one of the most charismatic and unmistakable animals in the sea. This enormous filter-feeding elasmobranch, with its unique checkerboard pattern of spots and stripes, inhabits all tropical and warm temperate seas [1]. Whale sharks are vulnerable to over-exploitation due to their conservative life history, slow swimming speed and docility at the surface, and highly migratory nature [2]. This species is listed as “Vulnerable” in the IUCN Red List of Threatened Species [3] and is one of eight shark species currently listed on Appendix II of the Convention on International Trade in Endangered Species of Wild Fauna and Flora (CITES).

Whale sharks travel long distances and the timing of their movements are typically associated with localized blooms of planktonic organisms and water temperature changes [1]. The use of both pop-up satellite archival tags (PSATs) and near-real-time satellite tags (e.g. Smart Position or Temperature Transmitting satellite tags, SPOTs) has greatly expanded our understanding of the broad horizontal movements of *R. typus* in certain parts of its range. In the Gulf of California (GOC), Eckert and Stewart (2001) [4] used towed satellite tags to demonstrate extensive movement of whale sharks into the North Pacific Ocean. Using towed tags off Southeast Asia, Eckert et al. (2002) [5] reported two whale sharks that traveled 4,567 and 8,025 km with an overall mean travel rate of 24.7 km day^−1^. By applying PSATs to whale sharks at Ningaloo Reef, Western Australia, Wilson et al. (2006) [6] documented long-term movements characterized by both inshore and offshore habitat utilization, northeasterly travels into the Indian Ocean, and vertical movements to at least 980 m. Collectively these studies indicate that *R. typus* is capable of transoceanic movements crossing numerous geopolitical boundaries, which highlights the need for both regional and multinational levels of management for this species. Whether these migrations (i.e. the seasonal movements of animals from one region to another) of several thousand kilometers are solely driven by feeding events or linked to other aspects of their life history is yet to be determined.

Although *R. typus* has been documented in various parts of the Gulf of Mexico (GOM) [7], [8] and Caribbean Sea [9], [10], we know very little about the movement and migration patterns of this species within and between these areas. Conventional tagging efforts off Gladden Spit, Belize has resulted in very few resightings outside the study area [11]. These authors did report the movements of one shark north to the Yucatan Peninsula and another migrating south near Utila, Honduras, about 570 and 112 km from the Gladden Spit tagging site, respectively. In a study using a towed satellite tag, Gifford et al. (2007) [12] demonstrated the northward movements of a single animal from Utila to a position in the central GOM.

In this paper we report results from the application of conventional and satellite tags to whale sharks off the northeastern corner of the Yucatan Peninsula (YP) in Quintana Roo, Mexico. Our study also used photo-identification as a non-invasive means of identifying individual sharks by their unique patterns of spots and scars, a procedure that has been successfully applied to *R. typus*. The overall objectives of this research included: 1) characterizing the size and sex composition of the whale sharks aggregating in the YP study area; 2) understanding residence time and site fidelity of the sharks utilizing this area each summer; and 3) tracking the sharks’ large-scale movements once they depart the YP feeding area.

## Methodology

### Ethics Statement

Research for this publication was carried out with prior permission from the Mexican federal government agency CONANP and was reviewed and approved by the Institutional Animal Care and Use Committee at Mote Marine Laboratory.

### Study Area

**Figure 1:**
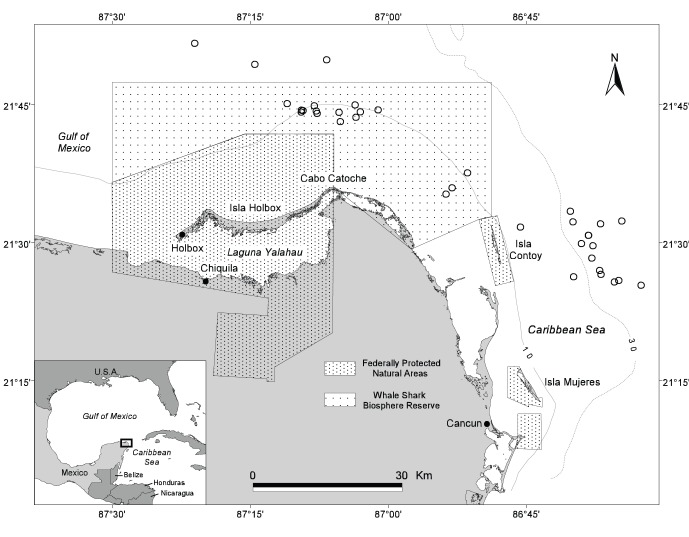
Whale shark study area off the northeastern Yucatan Peninsula, Mexico. The locations of PSAT tagging are indicated by open circles (n = 35).

Studies were conducted off the northeastern Quintana Roo portion of the YP from 2003–2012 (Fig. 1). This area’s hydrography is characterized by upwelling of subsurface water from the Caribbean Current onto the Yucatan Shelf [13], [14]. During spring and summer, cold, nutrient-rich, upwelled water intrudes over the Campeche Bank and creates a two-layered water column [13], [15]. There also is evidence that the westward-moving upwelled water contributes to the formation of a cyclonic eddy north of Cabo Catoche [13], [14] which leads to localized summer plankton blooms [16], [17]. Studies conducted since 2003 have shown the area to be a summer feeding ground for whale sharks in great numbers, with as many as 420 individuals observed in a single aerial survey [18]. Overall abundance has been estimated in one published study to be 521–809 individual sharks aggregating in the area primarily from May through September [19]. Mature and immature whale sharks of both sexes are present and all observable animals engage in various types of filter-feeding [20]. The study area is adjacent to federally protected natural areas that encompass land and coastal waters in the vicinity of Isla Holbox and Isla Contoy. In June 2009, as a result of research conducted by our group and others since 2003, a federal Whale Shark Biosphere Reserve was established to extend the marine protected area for the species (official decree available at www.conanp.gob.mx/sig/decretos/reservas/Tiburon.pdf). This now includes most of the whale sharks’ summer feeding grounds north of Isla Holbox and Isla Contoy (Fig. 1).

### Conventional Visual Tagging

From 2003 to 2011, external tags were used to identify individual whale sharks in the area as part of several research projects on feeding dynamics, genetics, growth, population size, and movement. These conventional visual tags consisted of sequentially numbered yellow laminated plastic placards (20×5 cm in 2003; 25×6 cm in 2004–06; 19×5.5 cm in 2007–11) attached to a modified stainless steel M-type dart (Type SSD; Hallprint Pty, Ltd, South Australia) by a monofilament tether. Visual tags were applied with a pole spear by a snorkeler to the shark’s left side just below the first dorsal fin. Resighting data were collected from field biologists working in the study area and through reports from snorkelers and divers observing whale sharks in Mexican waters and elsewhere. In addition, from 2004–09, visual tag resighting data were collected in interviews with whale shark ecotourism guides and operators based in Isla Holbox and Isla Mujeres.

### Photo-identification

Beginning in 2005, photographs were taken routinely by the research team using underwater digital cameras (Sony Cybershot P-93, Nikon Coolpix 4600) to document the pattern of spots on the sharks’ left sides, posterior to the fifth gill slit and forward of the first dorsal fin. This technique has been used successfully to identify individual whale sharks in a number of other studies [11], [21]–[25]. Secondary identification features, such as fin and body scars, also were photographed. Whale sharks in the YP area frequently showed wounds and scars consistent with injuries from boat propellers or hulls. In many cases the fins, especially the first dorsal and upper lobe of the caudal, were sliced or even missing. These features were photographed and noted to assist with identification of individual sharks. Photographs were submitted to the ECOCEAN Whale Shark Photo-identification Library (www.whaleshark.org). This online public database utilizes both the Interactive Individual Identification System (I^3^S) [26] and Modified Groth [22] pattern recognition algorithms for computer-assisted identification of whale shark photographs, which are submitted by researchers and the lay public around the world. Photo-matching can confirm movements of an individual shark from one region to another as well as residence time and return of sharks to the same area.

### Satellite Tagging - PSATs

Between August 2003 and September 2012, a total of 35 whale sharks were tagged with PSATs (1 PAT2, 6 PAT4, 28 Mk10-PAT; Wildlife Computers, Redmond, WA, USA) off Isla Holbox and Isla Contoy during July, August, and September. The tags archived ambient temperature, pressure, and light level measurements while attached to the animal for a user-set duration of 30–200 days. These variables were measured at regular intervals (ranging from every 3 to 60 seconds) and, due to limited bandwidth, summarized into time periods of either four, six, or eight hours to facilitate data transmission. Once detached and at the sea surface, the tags transmitted summaries of the archived data through the Argos satellite system. Physically recovered PSAT tags enabled retrieval of their full archived data.

Each PSAT was attached to a stainless steel dart (Type SSD; 34×8.5 mm; Hallprint) with a 15 cm tether comprising segments of 68 kg monofilament leader (Ande Inc., West Palm Beach, FL, USA) and 55 kg coated wire (Berkley, Spirit Lake, IA, USA). The monofilament portion of the tether was threaded through a pressure-activated guillotine designed to sever the tether if exposed to extreme depth (RD1500 in 2003, RD1800 after 2003; Wildlife Computers). The tether, excluding the RD device, was protected and color-coded with heat-shrink tubing (3M Electronics/Electrical, Austin, TX, USA). During the 2008–12 field seasons, antifouling paint (MDR-720 Transducer Paint, Marine Development and Research Corp., Merrick, NY, USA) was applied to the PSATs excluding the sensors and labeling. The float section of each tag displayed reward and contact information to augment the manufacturer’s tag number and contact information on the tag’s label.

Geographic positions at tagging were determined by a handheld global positioning system unit (Garmin GPSMAP® 276C). The pop-up positions of reporting PSAT tags were established as the first point of transmission with an Argos location class of 3, 2, or 1, indicating an accuracy radius within 1.5 km [27]. Back-calculated daily geolocations of each shark’s track from tagging to pop-up were first estimated using the manufacturer’s proprietary software (WC-GPE v. 1.02.005; Wildlife Computers). During this process, all light level data were inspected visually and conspicuously poor light curves were rejected and not used in subsequent analyses, as per the tag manufacturer’s software guidelines. Longitude estimates indicating travel distances that were unreasonably far from the preceding/following day’s estimate were considered as outliers and also were removed prior to estimating latitudes. We then used the state-space unscented Kalman filter (UKFSST) [28], an add-on package for the statistical environment R [29], and estimated movement parameters and predicted the most probable track (MPT) from the raw geolocations and tag-measured sea surface temperatures (SST). Following state-space estimation, we applied a secondary bathymetric correction that constrained estimated locations based on daily maximum depths that the shark achieved [30]. Estimates of MPT total distance were calculated using GE-Path software (v. 1.4.5). Lastly, we used the confidence intervals associated with our finalized tracks to identify core areas of whale shark activity by generating utilization distributions [30], [31] using the analyzepsat package for R [32].

### Satellite Tagging - SPOTs

In 2009, two sharks were double-tagged with PSAT and SPOT (SPOT5; Wildlife Computers) satellite tags. SPOT tags contain a saltwater switch that activates the tag when above the water’s surface and enables it to transmit a coded data stream to an orbiting satellite. The Argos Centers calculate the transmitter’s position by measuring the Doppler shift of its transmit frequency. Each position is coded with a location class (LC = 3, 2, 1, 0, A, B) with LC = 3 having the highest accuracy with an estimated error of <250 m [27]. One SPOT was attached as a towed tag and the other as a fin mount. The towed tag was secured with a 106 cm wire tether anchored by a steel M-type dart as described earlier. The fin-mounted tag was attached to the leading edge of the first dorsal fin using an experimental harness comprising neoprene and two rare earth magnets positioned on both sides of the fin, creating a strong but removable clamp. Mean daily locations from the SPOT tags (LC≥1) were compared to the raw light-derived PSAT geolocations, the uncorrected positions from the UKFSST model, and the bathymetrically corrected positions (the finalized MPT) for corresponding days. SPOT tag fixes were taken to be the true locations of the shark. PSAT error was quantified in degrees as the root mean square (RMS) error as described by Teo et al. (2004) [33] and previously used in this context by Hunter et al. (2003) [34]. For days where more than one SPOT position was available (LC≥1), daily mean latitude and longitude were used in the RMS calculation. Movement rate was calculated by taking the average speed among successive SPOT tag fixes (LC≥1).

### General Tagging Procedures

From a research vessel ranging 6–8 m in length and using information from other on-water and/or aerial surveys conducted concurrently, we located sharks on the surface by visually scanning for dorsal and caudal fins. When a surface-swimming shark was spotted and selected for tagging, the research vessel was maneuvered to drop off one or two snorkelers in the water just ahead of the moving shark. Using a pole spear, the tagger inserted the dart through the shark’s skin, anchoring it several centimeters into the subdermal tissue just below the first dorsal fin. In most cases, the tagger manually tugged on the inserted tag to ensure that it was well-seated. The non-tagging snorkeler dove below the pelvic region of the shark to identify sex by the presence or absence of male claspers. Maturity of males was assessed where possible. Mature males had elongated, differentiated claspers that extended beyond the pelvic fins, often with knobby ends; immature males had short, smooth claspers that did not extend beyond the pelvic fins [35]. Although female maturity could not be assessed for certain, the pelvic region was observed for signs of obvious, significant swelling possibly indicative of pregnancy. Each shark’s total length was estimated to the nearest 0.5 m by maneuvering the vessel alongside the animal and comparing its head and tail positions to measured marks on the vessel’s gunwale. Every effort was made to obtain a length estimate when the shark was perfectly parallel with the vessel. Because the sharks did not necessarily swim straight at the water’s surface, but often swam with the tail at a somewhat lower depth than the head, any associated error with this technique would be skewed towards underestimating the shark’s length. For each satellite tag deployment (and periodically during conventional tagging), water quality parameters (temperature, salinity, dissolved oxygen) were measured with a handheld electronic meter (YSI 85, YSI Inc., Yellow Springs, OH, USA) at the water’s surface, mid-depth, and at the bottom or maximum measurable depth of the meter’s limited cable (30 m). Additionally, Secchi depth (20 cm disk diameter) was recorded for most satellite tag deployments.

## Results

### Conventional Tagging

**Figure 2:**
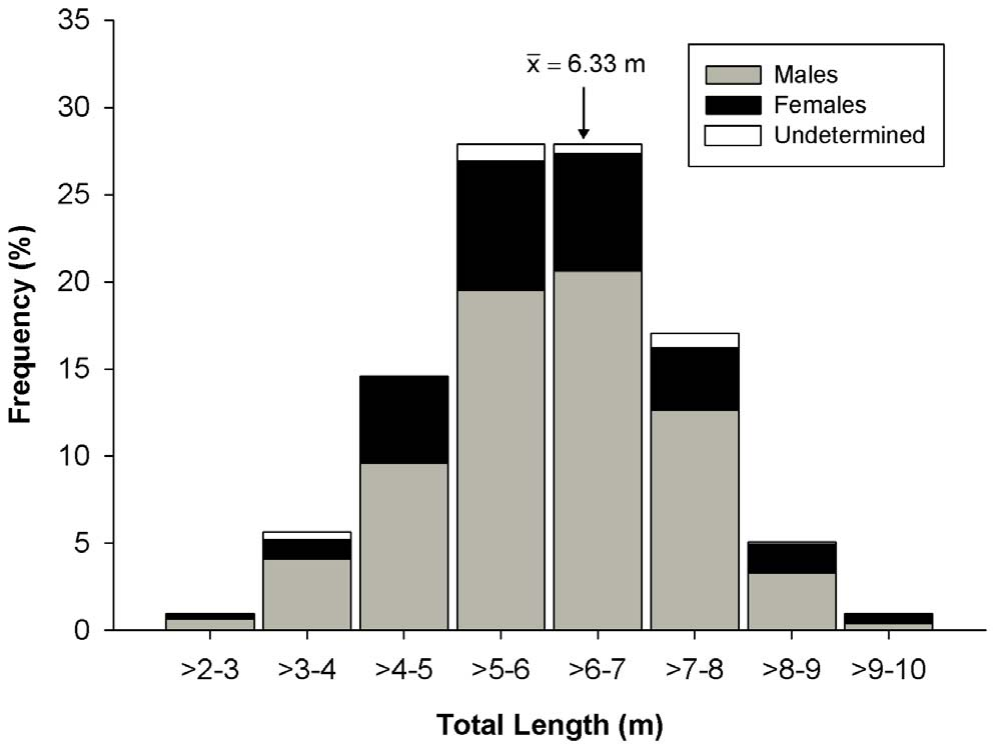
Length-frequency histogram of conventionally tagged whale sharks of known length in the Yucatan study area 2003–2011 (n = 728).

A total of 813 whale sharks were tagged with conventional tags between 2003 and 2011 (Table 1). The sex ratio and size range of tagged animals remained relatively consistent over the years of the study. The majority of tagged sharks were males, with an overall M:F ratio of 2.6∶1. Total length (TL) averaged 6.33 m (SD = 1.31) and ranged 2.5–10 m. Juveniles, subadults, and sexually mature animals of both sexes (males and females averaged 6.34 m and 6.30 m TL, respectively) were tagged (Fig. 2). A total of 1,421 resightings of these tagged sharks were documented, 348 by project biologists working in and near the study area and 1,073 resightings reported by local whale shark ecotourism operators (Table 2; numbers of “resightings” include sightings of the same shark on different days). Although the majority of the resighted sharks had been tagged the same year (80.2%), 17.0% of the resighted animals had been tagged one year prior and 2.8% were sharks tagged two or more years earlier, with four years as the longest duration between tagging and resighting. Some sharks were observed with only the tag’s tether remaining in the skin but with no placard attached, indicating the sharks had previously been tagged and the placard was lost due to fouling, wear or other factors. Overall, 83 of the tagged sharks were resighted in the study area in consecutive years while four of these sharks were resighted off the YP in three consecutive years. Tagged sharks also were reported by sport fishermen and recreational divers in other locations. Most notably, a 7 m male tagged 6 July 2008 was resighted 98 days later off St. Petersburg, Florida, USA, a straight-line distance of 791 km (photo-identification revealed this same shark was observed off the YP during the summers of 2010 and 2011). Additionally, two male whale sharks tagged off the YP in August, one in 2003 and the other in 2006, were reported near the island of Utila, Honduras in October (in the same respective years), a distance of approximately 610 km. Conversely, whale sharks tagged off Honduras by other researchers have been observed off the YP. For example, a 6.5 m female *R. typus* tagged 22 April 2005 off the north shore of Utila (Whale Shark & Oceanic Research Center [WSORC] tag #U603) was resighted by a Mexican ecotourism operator 30 days later north of the YP off Cabo Catoche, 630 km from the tagging site.

**Table 1:** Summary of whale shark (*Rhincodon typus*) conventional tagging off the northeastern Yucatan Peninsula.

	Total			Unknown	Ratio	Est. Total
Year	Tagged	Males	Females	Sex	M:F	Length (m)
2003	17	9	5	3	1.8∶1	3.5–10.0
2004	172	113	39	20	2.9∶1	3.0–9.0
2005	164	105	50	9	2.1∶1	2.5–9.5
2006	201	140	51	10	2.7∶1	3.5–8.0
2007	68	47	16	5	2.9∶1	3.5–9.0
2008	99	72	26	1	2.8∶1	3.0–9.0
2009	85	60	21	4	2.9∶1	2.5–8.5
2010	6	1	5	0	0.2∶1	7.0–8.5
2011	1	0	1	0	0∶1	7.5
**Totals**	**813**	**547**	**214**	**52**	**2.6∶1**	**2.5–10.0**

**Table 2:** Summary of whale shark conventional tag resightings reported by project biologists and whale shark ecotourism operators within the study area.

					No. Individuals by Tagging Year					
	Cumulative	Total No.	Biologist/Ecotour	No. Individuals	Same	1 Yr	2 Yrs	3 Yrs	4 Yrs	5–8 Yrs
Year	No. Tagged	Resightings	Resightings	Resighted	Yr	Prior	Prior	Prior	Prior	Prior
2003	17	0	0/0	0	0	–	–	–	–	–
2004	189	101	71/30	67	66	1	–	–	–	–
2005	353	252	32/220	102	93	9	0	–	–	–
2006	554	332	69/263	177	150	23	4	0	–	–
2007	622	203	9/194	78	42	33	2	1	0	–
2008	721	385	49/336	129	97	24	6	1	1	0
2009	806	135	105/30	84	66	16	2	0	0	0
2010	812	12	12/0	9	4	4	1	0	0	0
2011	813	1	1/0	1	1	0	0	0	0	0
**Totals**	**813**	**1,421**	**348/1,073**	**647**	**519**	**110**	**15**	**2**	**1**	**0**

To assess residence time, we examined resightings data for whale sharks seen in the study area during the same year of tagging (with ≥1 day between tagging and resighting). Mean duration between tagging and the last resighting in a given year was 26.4 days (SD = 24.3; range = 1–86 days; n = 177) for biologist resightings and 21.9 days (SD = 18.0; range = 1–106 days; n = 282) for ecotourism industry resightings (overall mean = 23.6 days).

### Photo-identification

As of 5 March 2013, the ECOCEAN database documented 956 uniquely identified whale sharks through photo-identification in the YP area. Of these sharks, 40 were identified for the first time in 2012 and hence were not considered in determining resighting proportions. Of the remaining 916 sharks, 528 (57.7%) were observed in the YP only during a single year. Of the 388 whale sharks resighted in multiple years, 156 (17.0%) were observed during two consecutive years, 67 (7.3%) in three consecutive years, 42 (4.6%) in four consecutive years, four in five consecutive years (0.4%) and two sharks (0.2%; ECOCEAN IDs MXA-040 and MXA-342) were observed off the YP in six consecutive years. The remaining 117 individuals (12.8%) were sharks with 2–7 years between YP resightings.

Several of the whale sharks photo-identified off the YP also were resighted in other areas including off the coasts of Honduras (26 individuals), Belize (14), Louisiana (4), west Florida (2), and east Florida (1). The majority of sharks identified off Honduras and Belize in the northwestern Caribbean Sea were seen during winter and early spring (December-April); resightings off Louisiana in the northern GOM occurred in June and September; Florida resightings in the eastern GOM occurred in July/August and October; and the Florida observation in the northwestern Atlantic Ocean was in late December. The most notable movement between areas was demonstrated by a subadult male whale shark (MXA-008; nicknamed “Sinaloa”) whose 21 documented ECOCEAN encounters spanning eight different years demonstrated movements among Utila Island in Honduras (October), Gladden Spit in Belize (April and June), and the YP in Mexico (July-September). Several of the sharks identified through photo-identification also had reports of conventional visual tags and/or fin wounds which further corroborated and confirmed their photo-identifications.

As with the conventional tagging results, we utilized photo-identification data to gain insight into whale shark residence time in the summer feeding grounds by considering just the sharks resighted more than once off the YP during a single year (with ≥1 day between sightings). Mean duration between first and last resighting by photo-identification in a given year was 32.5 days (SD = 30.5 days; range = 1–187 days; n = 456).

### PSAT Tagging

Satellite-tagged sharks ranged 4.5–9.0 m TL and comprised 22 females, 12 males, and one of undetermined sex (Table 3). At satellite tagging locations (Fig. 1) water depth ranged 8–43 m, Secchi depth ranged 5–28 m, and SSTs and salinities ranged 24.0–30.0°C and 33.5–35.8 ppt, respectively. We received data from 28 of the 35 PSATs (ranging 2–190 days at large with tag attached), while the other seven failed to report (20%). The tags transmitted from a broad range of geographic areas including the western, central, and eastern GOM, the Caribbean Sea, and one from the mid-Atlantic Ocean, south of the equator. Eight tags were physically recovered and downloaded; of these, three tags (Shark # 4, 10 and 18) washed ashore in Texas, two (6 and 7) were prematurely removed through human interference at the YP site shortly after tagging, another (33) was recovered after its premature release in the YP area, one was recovered near Miami, Florida, USA (30), and one was discovered in the Bahamas more than five years after tagging (9). Only five of the 28 reporting tags (18%) began transmitting their archived data precisely on their programmed pop-up dates, while the others reported either early or after the specified date. In seven of the late-reporting tags, the PSATs popped up on the programmed date but data transmission was delayed for 6–44 days (Sharks 10, 11, 13, 14, 22, 23 and 30) and thus the precise pop-up location could not be determined. In these instances, the UKFSST model was instructed to incorporate this end-point uncertainty into its calculations of the MPT (i.e. *fix.last = FALSE*) and essentially iterate the pop-up position (Table 3).

**Table 3:** Summary of 35 PSAT tags deployed on whale sharks (*Rhincodon typus*) off the northeastern Yucatan Peninsula.

Shark	PSAT		Est.	Tagging			Programmed	Pop-up			Days of	Distance	Max.
No.	Version	Sex	TL (m)	Date	Lat (°N)	Long (°W)	Duration (d)	Date	Lat (°N)	Long (°W)	Data	(km)^†^	Depth (m)
1	PAT2	U	7–8	8/13/03	21.59	86.89	92	Failed to Report			–	–	–
2	PAT4	M	4.5	8/10/04	21.74	87.06	92	Failed to Report			–	–	–
3	PAT4	F	6.5–7	8/12/04	21.63	86.85	90	Failed to Report			–	–	–
4*	PAT4	M	6	8/31/05	21.75	87.16	31	10/1/05	23.51	95.60	31	887	954
5	PAT4	M	5.5	9/2/05	21.60	86.88	30	10/2/05	19.42	87.40	30	260	304
6*	PAT4	M	5.5	7/20/06	21.84	87.11	30	7/22/06	21.50	87.05	2	38	11
7*	Mk10	F	7	7/21/06	21.75	87.16	100	8/8/06	21.52	87.37	18	34	17
8	Mk10	M	6.5	7/21/06	21.76	87.14	150	Failed to Report			–	–	–
9*	Mk10	F	8.5	7/22/06	21.75	87.02	200	1/28/07	25.11	87.52	190	377	1,504
10*	Mk10	M	7	7/23/06	21.76	87.19	120	11/20/06	28.5	94.39	120	1,041	1,530
11	Mk10	F	7	9/13/06	21.43	86.56	60	11/12/06	25.19	94.87	60	945	1,376
12	PAT4	M	8	9/13/06	21.43	86.57	30	10/17/06	25.28	92.91	34	775	464
13	Mk10	F	8	9/14/06	21.42	86.52	100	12/21/06	22.21	87.87	98	165	1,072
14	Mk10	M	7.5	8/6/07	21.73	87.09	120	12/4/07	20.21	88.51	120	224	1,720
15	Mk10	F	7.5	8/29/07	21.84	87.25	150	1/26/08	−1.82	25.27	150	7,213	1,600
16	Mk10	F	5	8/30/07	21.88	87.36	90	11/28/07	19.54	91.38	90	550	1,400
17	Mk10	M	7.5	8/8/08	21.74	87.13	180	11/13/08	18.81	81.18	97	700	1,432
18*	Mk10	F	9	8/8/08	21.75	87.09	120	8/11/08	21.84	87.26	3	20	8
19	Mk10	F	6.5	8/8/08	21.75	87.13	150	Failed to Report			–	–	–
20	Mk10	F	8	8/9/08	21.76	87.06	180	11/17/08	23.81	84.10	100	379	1,528
21	Mk10	M	7	8/10/08	21.75	87.05	150	1/7/09	13.57	80.50	150	1,143	1,560
22	Mk10	F	8	8/11/08	21.75	87.16	120	12/9/08	25.28	93.82	120	784	1,392
23	Mk10	F	7.5	7/13/09	21.54	86.65	180	1/9/10	22.76	85.77	180	163	1,240
24	Mk10	F	7.5–8	7/13/09	21.56	86.66	180	10/30/09	23.78	81.51	109	583	1,368
25	Mk10	M	7	7/16/09	21.54	86.56	180	9/14/09	24.50	89.32	60	433	1,888
26	Mk10	F	6.5–7	7/16/09	21.54	86.60	180	10/30/09	24.53	93.61	106	791	1,008
27^DT^	Mk10	F	7.5	8/28/09	21.44	86.65	150	12/18/09	23.28	86.20	112	197	1,672
28^DT^	Mk10	F	8	8/29/09	21.50	86.63	150	12/6/09	23.25	84.79	99	281	1,392
29	Mk10	F	8–8.5	7/28/10	21.50	86.61	210	Failed to Report			–	–	–
30*	Mk10	F	8	7/28/10	21.51	86.62	180	1/24/11	23.15	88.77	180	286	1,408
31	Mk10	F	7.5	7/28/10	21.51	86.62	150	8/31/10	22.60	86.70	34	121	80
32	Mk10	F	7.5–8	7/29/10	21.44	86.60	180	9/2/10	22.41	86.97	35	112	72
33*	Mk10	F	8.5	7/29/10	21.45	86.60	150	9/24/10	22.08	87.11	57	87	116
34	Mk10	F	8	7/17/11	21.47	86.61	180	Failed to Report			–	–	–
35	Mk10	M	8.5	9/23/12	21.53	86.75	180	10/26/12	14.39	81.14	33	990	928

U = Undetermined; ^†^ Minimum at-sea distance from tagging location to pop-up location (actual or iterated); * Tag recovered; ^DT^ Shark double-tagged with SPOT5.

The longest at-sea straight-line distance between tagging and pop-up was 7,213 km for a 7.5 m female (Shark 15) at large 150 days (Table 3). This distance represents a minimum geographic displacement between start and stop of data recording and is unquestionably an underestimate of actual distance traveled by the shark, as it does not take into account forays off a straight path or diving excursions. The maximum depth recorded for any shark in this study was 1,888 m by a 7 m mature male (Shark 25) on 14 September 2009 in the central GOM, north of the YP (Table 3).

Of the 28 tags reporting, raw geolocations from 22 tags demonstrating movements away from the study site were analyzed using the UKFSST model. The remaining six tags (from sharks at large 2–57 days) had raw geolocations and vertical movement profiles indicating they had not migrated appreciably away from the tagging area and hence those data were not suitable for UKFSST analysis. The values of the model’s estimated parameters are presented in Table 4. Of the directed movement parameters (*u* and *v*) that were estimated, the median values (in nautical miles [nm] day^−1^) were −0.19 and 0.11, respectively. The diffusion estimate (*D*) varied broadly (11.79–3,365.26 nm^2^ day^−1^) and had a median value of 449.98 nm. The median error associated with the estimates of longitude (σ_x_) and latitude (σ_y_) along the MPTs were 0.16° (10 nm) and 1.19° (72 nm), respectively. The smoothing radius (*r*) was only estimated by the model for one of the tracks (Shark 35) but instead fixed for the others to optimize the fit (i.e. minimize the negative log-likelihood [log L] value) and/or facilitate data convergence and ranged 93–275 nm. Based on the estimated MPTs, the sharks moved at a mean horizontal velocity of 28.56 km day^−1^ (SD = 13.715), or 1.19 km hr^−1^.

**Table 4:** Estimated parameters for PSAT tags analyzed with UKFSST and associated mean speeds from the predicted most probable track (MPT).

								Track	Est. Total	Est. Mean
Shark No.	*u*	*v*	*D*	*σ_x_*	*σ_y_*	*r*	log L	Duration (d)	Distance (km)	Rate (km/day)
4	0	2.77	1,042.37	0.25	1.14	250	97.84	31	1,450.83	46.80
5	1.00	−4.41	11.79	0.28	0.48	160	65.72	30	295.19	9.84
9	−0.01	0.93	319.72	0.21	1.25	160	599.71	190	3,893.27	20.49
10	3.59	0	776.52	0.1	1.97	250	421.28	120	3,488.85	29.07
11	0	29.76	3,218.97	0.1	1	95	119.60	60	2,793.61	46.56
12	11.11	10.33	192.75	0.18	2.06	93	105.67	34	927.86	27.29
13	0.30	−0.21	374.66	0.1	2.24	155	164.02	94	2,078.84	22.12
14	−0.21	−0.11	449.98	0.41	1.11	248	186.61	117	1,620.24	13.85
15	−24.42	−10.33	793.27	0.48	2.08	275	219.14	150	7,771.67	51.81
16	2.54	−2.38	643.79	0.22	1.33	200	268.39	90	3,875.22	43.06
17	−3.46	−1.58	316.41	0.1	1.08	235	317.81	96	3,217.77	33.52
20	−1.61	1.46	342.51	0.11	1.04	145	343.23	100	2,606.02	26.06
21	−1.43	−5.93	1,105.62	0.10	1.76	179	212.09	150	4,627.94	30.85
22	0	0	572.82	0.1	1.00	148	299.81	120	3,370.62	28.09
23	−0.29	0.49	223.13	0.26	0.88	188	246.46	180	1,617.03	8.98
24	−2.20	2.85	460.13	0.36	1.28	175	172.45	109	1,870.46	17.16
25	9.52	−1.37	439.87	0.05	0.42	155	150.34	60	1,525.62	25.43
26	3.90	3.04	218.90	0.19	0.78	160	283.50	106	1,910.35	18.02
27	−0.19	−0.24	429.34	0.12	1.29	175	329.65	112	3,071.28	27.42
28	−1.49	0.87	420.18	0.39	1.85	170	205.00	99	1,966.71	19.87
30	0.42	0	492.48	0.1	1	200	347.48	180	3,766.07	20.92
35	−29.40	−43.18	3,365.26	0.15	1.81	190.6	69.72	30	1,834.16	61.14

Directed movement parameters (*u* and *v)* expressed in nm day^−1^; diffusion estimate (*D)* in nm^2^ day^−1^; error estimates of longitude and latitude (σ_x_ and σ_y_) in degrees; smoothing radius (*r*) in nm. Negative log-likelihood values (Log L) are a measure of the model’s fit (smaller value = better fit). Values of 0 (for *u* and *v*) indicate models in which those parameters were not active. Values of 0.1 (for *σ_x_*) and 1 (for *σ_y_)* indicate models in which those parameters assumed UKFSST default values. Smoothing radius (*r*) was fixed, with the exception of Shark 35, to optimize the model’s fit. Reynolds Optimally Interpolated SST was used as the SST field in all cases.

**Figure 3:**
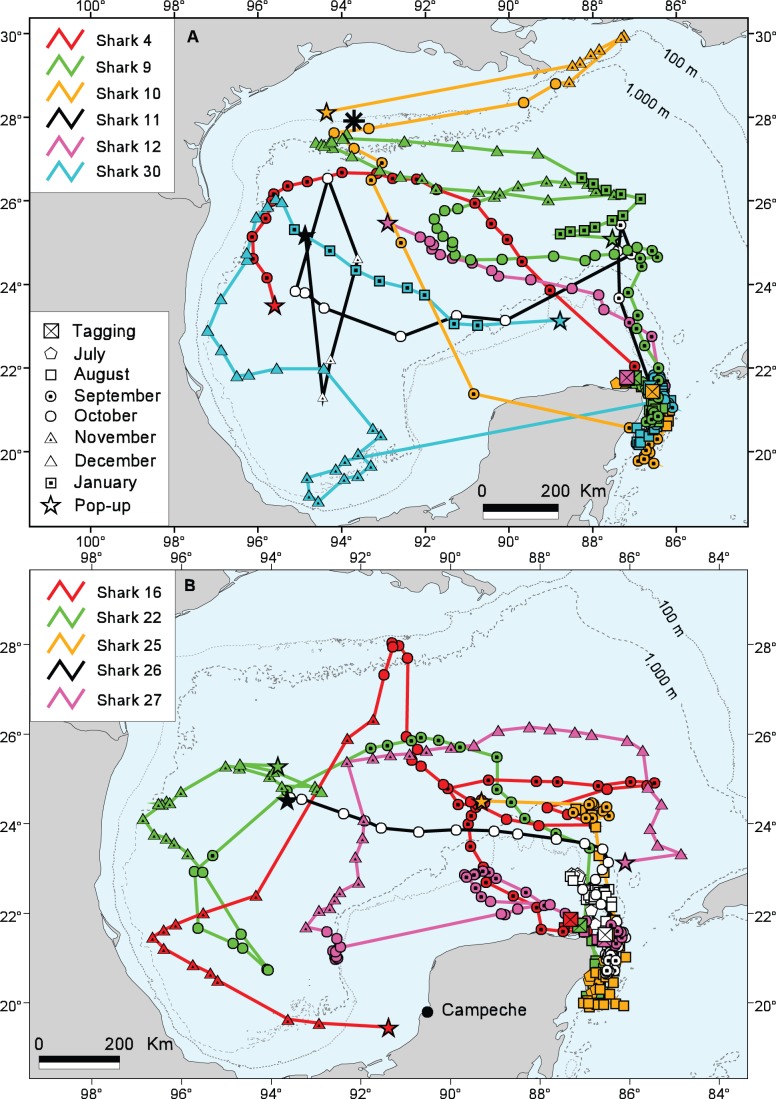
Most probable tracks of whale sharks moving into the Gulf of Mexico. (A) Sharks 4, 9, 10, 11, 12, and 30. (B) Sharks 16, 22, 25, 26, and 27. The location of the Flower Garden Banks in the northwestern Gulf are indicated by a black asterisk in 3A.

**Figure 4:**
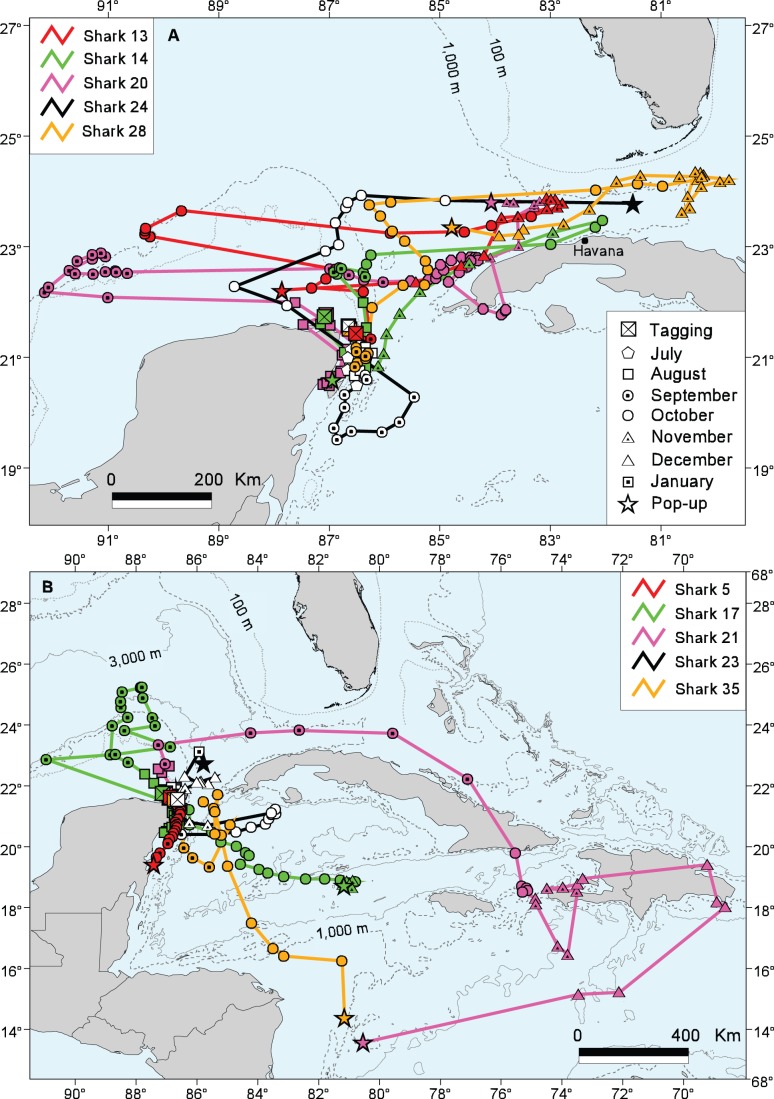
Most probable tracks of whale sharks moving into the vicinity of the Straits of Florida and Caribbean Sea. (A) Sharks 13, 14, 20, 24, and 28. (B) Sharks 5, 17, 21, 23 and 35.

**Figure 5:**
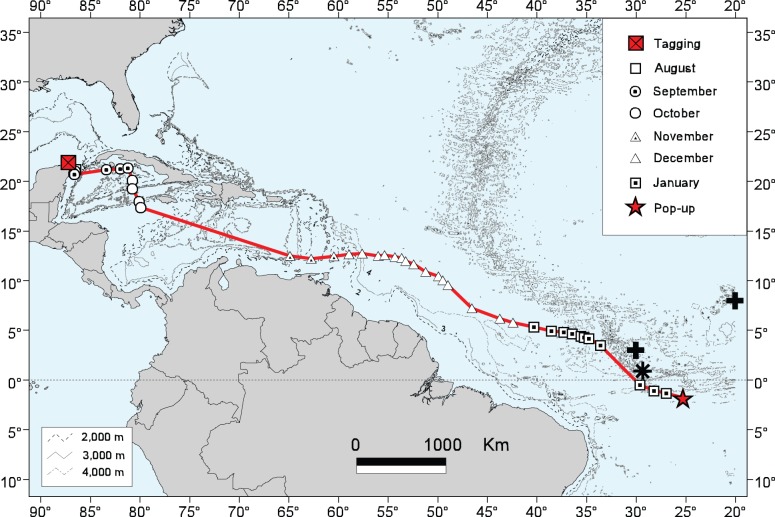
Most probable track of Shark 15 (“Rio Lady”) derived from the unscented Kalman filter (red line). The locations of the Saint Peter and Saint Paul Archipelago (black asterisk) and very small whale sharks (black plus signs) as reported by Kukuyev (1996) [93] are indicated near the tag’s pop-up location (lat/long for newborn shark data points from Martin (2007) [90]).

All satellite-tagged sharks remained in the vicinity of the study area until late August to early October (Figs. 3–5). We group their subsequent movements into three zones: primarily into the Western GOM; primarily into the Eastern GOM and Straits of Florida; and primarily into the Caribbean Sea, with one whale shark continuing to the South Atlantic Ocean.

#### Movements into the Western GOM

Upon leaving, nine animals (Sharks 4, 9, 10, 11, 12, 16, 22, 25 and 26; 4 males and 5 females) moved in a north-to-northwesterly direction into the central GOM before moving toward the western GOM; only two animals departed with a westerly track (27 and 30) (Figs. 3A, 3B). Shark 4 moved northwest across the GOM during its 31-day track, a migration demonstrating one of the highest mean rates of movement for any animal in this study at 46.80 km day^−1^ (Table 4). Shark 9 moved north from the study area by mid-September and occupied the central GOM for most of October and November before migrating into the northwest GOM in early December. During the next 2–3 weeks this shark remained along the continental slope off Texas, in the general vicinity of the Flower Gardens Bank, before moving in January into the central GOM where its tag popped up prematurely after 190 days. Shark 10 departed the study area in mid-September in a northwesterly direction and reached the shelf edge waters south of Sabine Pass, Texas by the beginning of October. This shark spent the first half of November in the continental slope waters east of the Mississippi Delta, showing the farthest northward movement of any shark in the study, before its tag popped up at a location south of Galveston, Texas in late November. Shark 11 moved north from the study site and then traveled into the west-central GOM by early October, into the northwest GOM off Texas by late October, and into the south-central GOM by early November. Shark 12 moved in a northwesterly direction from the study area before its tag popped up in the west-central GOM, 34 days after tagging. Shark 30 departed the study area in late October, remaining in the area later than any other of the satellite-tracked sharks, and moved into the southeast GOM by November, into the western GOM by December, and then began migrating eastward in January when its tag popped up in the south-central GOM, north of the YP. Shark 16 moved into the northern GOM by late October, migrated into the southwestern GOM in early November, then traveled into southern GOM waters where its tag began transmitting in late November, about 90 km southwest of the city of Campeche, Mexico (Fig. 3B). Shark 22 moved into the central GOM in September, migrated into the southwest GOM by mid-October before moving north again into the west central GOM by early November. Shark 26 remained in the general area of the YP until early October and then migrated north before moving into the west-central GOM. Shark 27 left the study area in mid-September, moving in a westerly direction into shelf-edge waters west of the YP where it remained until early November. This shark then moved northerly into the central GOM by late November, then easterly in early December to just off Florida’s continental shelf, and finally southerly before its tag popped up northeast of the YP about 200 km north of the study area. Shark 25 remained in the vicinity of the study site for about one month after tagging and then moved north and off the Yucatan shelf, where its PSAT prematurely popped up in late August, likely due to an extreme dive of 1,888 m that activated the pressure-sensitive guillotine and severed the tag’s tether. Although the guillotine is designed to cut at 1,800 m, deeper dive data are sometimes obtained. We recorded a dive of 1,928 m for a 7 m immature male whale shark in the northeastern GOM in August 2010; these data are not included here as this shark was not tagged at the YP study site.

#### Movements into the Eastern GOM and straits of Florida

Whale shark movements around the eastern GOM and Straits of Florida were seen in Sharks 13, 14, 20, 24, and 28 (Fig. 4A). After departing the study area in a northerly direction, Shark 13 moved westward to shelf edge waters by mid-October and then shifted eastward in late October toward the Straits of Florida, where it spent several weeks off the northwest coast of Cuba before migrating back to GOM waters north of the YP in mid-December. Shark 14 left the study area in late August and moved eastward into the Straits of Florida and along the northwest coast of Cuba by late October. This shark began moving westward toward the YP in early November, crossed the Yucatan Channel, and ended its 117-day track north of the island of Cozumel, Mexico in the Caribbean Sea. Shark 20 migrated west upon leaving the study area in late August and spent most of September in the shelf edge waters northwest of the YP. This shark then moved eastward toward the Straits of Florida in early October and remained in the vicinity of western Cuba until its tag popped up on 17 November, about 185 km northwest of Havana. Beginning in early August, Shark 24 moved southerly into the Caribbean Sea before heading back north off the YP tagging area by early October. This shark then migrated in an easterly direction into the Straits of Florida, where its tag detached but did not transmit for another 8 days until it washed ashore in Cuba about 55 km east of Havana (efforts to recover this tag in Cuba were unsuccessful). After departing the study area in late September, Shark 28 moved in a north- to-northeasterly direction before moving east into the Straits of Florida off north-central Cuba by the end of October. This shark remained in this area for the first three weeks of November before migrating west along Cuba’s northwest coast where its tag popped up after 99 days at liberty, about 248 km west-northwest of Havana.

#### Movements into the Caribbean sea and South Atlantic Ocean

Movements into the Caribbean Sea were seen in Sharks 5, 15, 17, 21, 23 and 35 (Figs. 4B, 5). The track of Shark 5 indicated a southerly migration relatively close to the Mexican Caribbean coast where its tag popped up after 30 days only 5 km from shore, near the mouth of Bahía del Espíritu Santo, Quintana Roo. Shark 17 moved north from the study area in September before beginning a southerly track into the Caribbean that ended mid-November about 305 km west-northwest of Jamaica. Shark 21 departed the study site in early September, migrated eastward through the Straits of Florida and along the north Cuban coast, and apparently entered the Caribbean Sea via the Windward Passage between Cuba and Haiti. This female remained in the vicinity of Hispaniola’s coastal waters from October through mid-December before migrating in a southwesterly direction toward Central America and ending its 150-day track about 325 km east of Nicaragua. Although Shark 23′s track had one of the longest durations of the study (180 days), the amount of data received was limited resulting in periods without daily raw geolocations and, therefore, we had difficulty generating a realistic MPT. But in general, this shark remained in the study area until late September when it moved south and then easterly to an area southwest of Cuba’s Isla de la Juventud. The shark remained in this vicinity for most of October before moving westerly back toward the YP by early November, remaining there for most of November before moving to the northeast between the YP and Cuba. Shark 35 moved southward soon after its late September tagging but then shifted back north into the Yucatan Channel between Cuba and Mexico. This shark then began moving in a southerly direction by mid-October before its tag detached early and ended its track about 222 km east of Nicaragua.

The longest movement by a whale shark in this study was by a female estimated to be 7.5 m TL (Shark 15; nicknamed “Rio Lady”), which traveled in a generally east-southeast direction through the Caribbean Sea and into the open Atlantic Ocean to a point just south of the equator and near the Mid-Atlantic Ridge, about 1,181 km off the northeast coast of Brazil, a track that took 150 days (Fig. 5). Based on the estimated MPT, this shark traveled a horizontal distance of 7,772 km during this period, which is the second-longest confirmed track recorded for a whale shark to date [36]. This distance required a minimum average speed of 52 km day^−1^, the second highest for any shark in this study (Table 4). At the time of tagging off Isla Holbox (29 August 2007), this female’s pelvic region was noticeably enlarged but we could not be certain of her maturity or reproductive condition. In July and August 2006, resightings of Rio Lady north of Cabo Catoche and Isla Contoy, respectively, were confirmed through ECOCEAN photo-identification. No evidence of her returning to the YP after 2007 was found until Rio Lady was resighted off Isla Mujeres in July 2011, when we attached a second PSAT to her (identified as Shark 34 in Table 1). Unfortunately this second tag did not report for unknown reasons. In summer 2012, sightings of Rio Lady in the YP study area were confirmed again through photo-identification.

#### Core-Use areas

**Figure 6:**
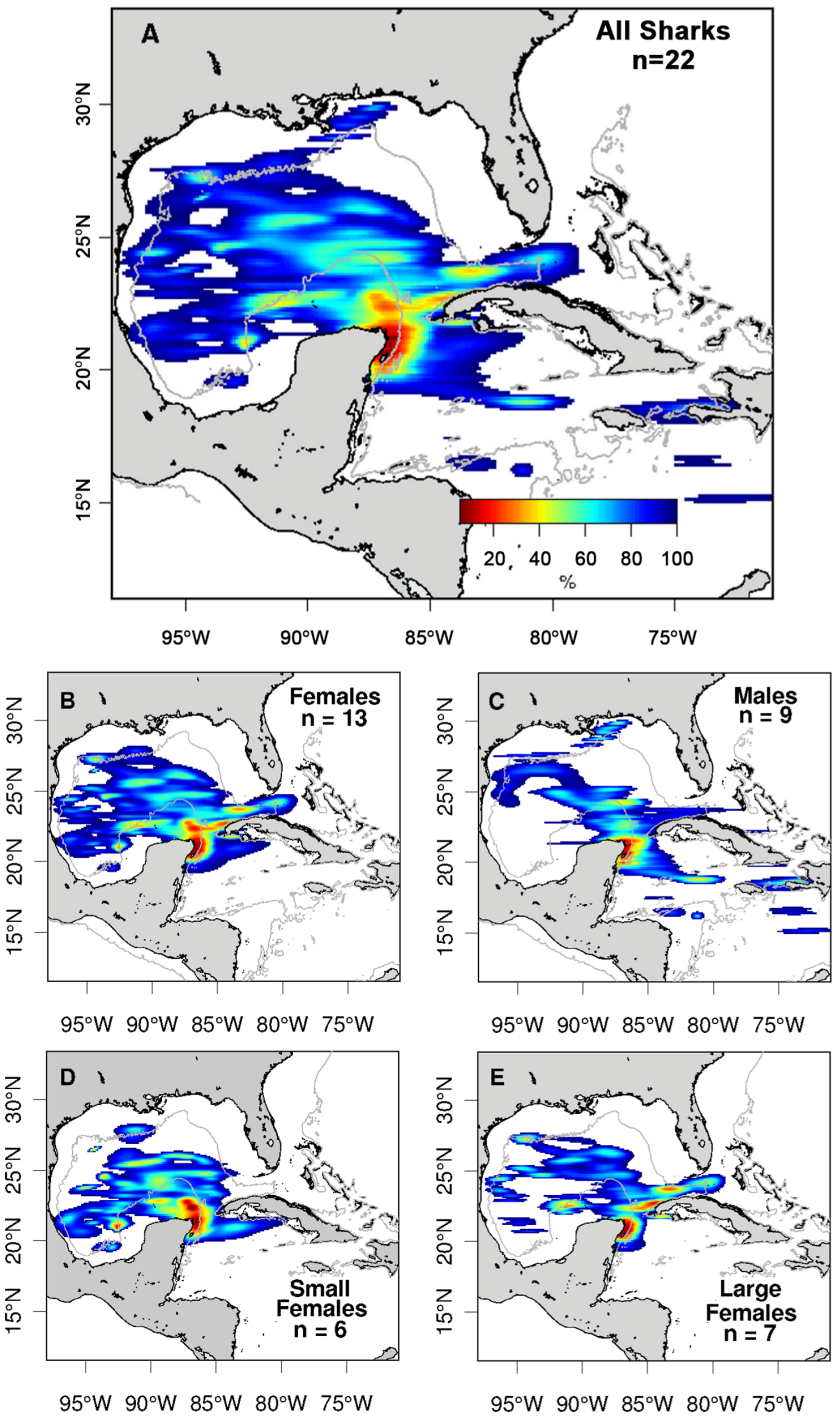
Habitat utilization distributions for satellite-tagged whale sharks based on their most probable tracks. (A) All sharks combined; (B) Females; (C) Males; (D) Smaller females (<8 mTL); (E) Larger females (≥8 m TL). The 1,000 m bathymetric contour approximates the shelf edge.

The habitat utilization distribution identified several whale shark core-use areas aside from the conspicuous Yucatan tagging area (Fig. 6). The combined distribution (all 22 tracks; Fig. 6A) showed a prominent core-use area immediately north of the tagging site, likely highlighting the primary route the sharks were taking upon exiting the summer YP feeding grounds. Other core-use areas included two areas off northwest Cuba, two areas west to northwest of the YP, and a small area in the northwestern GOM. These five areas all are found in shelf edge habitats near the 1,000 m contour line. A comparison of the distributions by sex (Figs. 6B–C) indicated that females utilized the core areas off northwest Cuba and west of the YP more than males. Separating the females by size (Figs. 6D–E) revealed that the larger females (≥8 m TL) used the area off NW Cuba more than the smaller females (<8 m TL), which appeared to utilize the shelf edge habitat west of the YP to a greater extent.

### Double-Tagging with PSAT and SPOT Tags

**Figure 7:**
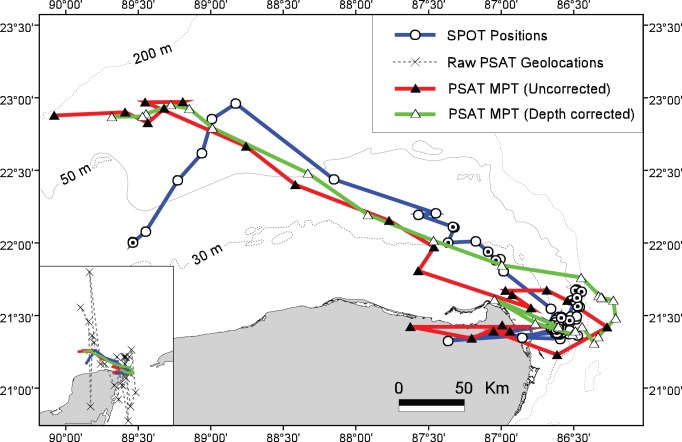
A comparison of the tracks from the double-tagging of Shark 27. The SPOT track (blue line) includes location qualities of 3, 2, 1, 0, and A. The highest quality SPOT locations (LC≥1) have a black dot inside their white circular symbol. The light-derived PSAT results as raw geolocations (broken line of inset figure), the uncorrected MPT (red line), and the bathymetrically corrected MPT (green line) are shown for the same time period as that of the SPOT tag.

Shark 27 was double-tagged with a PSAT and towed SPOT. The SPOT tag transmitted regularly for 30 days before it detached from the shark, as confirmed by this tag’s temperature and histogram data. The PSAT popped up prematurely from its programmed time (150 days) after 112 days at large. A comparison of this shark’s SPOT track (LC = 3, 2, 1, 0, and A) with the PSAT’s raw light-based geolocations, uncorrected MPT, and bathymetrically corrected MPT from the point of tagging (28 August, 2009) to the last usable SPOT location (27 September, 2009) is shown in Figure 7. In a quantifiable comparison with the SPOT locations (LC≥1), the RMS error for the latitude and longitude of the raw geolocations was 1.922° and 0.301°, respectively (n = 13). In a similar comparison, the RMS error for the uncorrected MPT was 0.271° for latitude and 0.476° for longitude (n = 13) while the error for the bathymetrically corrected MPT was 0.277° for latitude and 0.289° for longitude (n = 13). The average movement rate between successive SPOT fixes for Shark 27 was 1.93 km h^−1^. Shark 28 also was double-tagged with a fin-mount SPOT and a PSAT, however, the SPOT transmitted sporadically over a 36-day period and did not provide a useful track for comparison with the same shark’s PSAT.

## Discussion

### Evaluation of Methods

Conventional tags were the primary means of identifying individual whale sharks during the first six years of this project (2003–08) when the field work was confined to the waters north of Cabo Catoche and Isla Contoy (Fig. 1), areas with poor water visibility (Secchi depth ∼5–10 m). After 2008, the research focus shifted to the “Afuera” aggregation [18] where the exceptional visibility (Secchi depth ∼13–28 m) allowed photo-identification as the primary means of identifying individual sharks. As a result, more individual whale sharks have been documented through photo-identification at the YP site than at any other aggregation site in the world. The decision to switch to photo-identification was influenced by the low number of long-term resightings of conventional tags (only a single case ≥4 years at large), which was likely due to tag loss after the tags were attached for more than one year. Tag retention problems have been reported in other whale shark studies [11], [37] and the high shedding rates have been largely attributed to breakage of the tags’ plastic placards. In our study, however, the visual tags did provide the ability to readily identify individual sharks in the short term, which was highly useful at our study site with hundreds of sharks. External visual tags prevented the unnecessary resampling of the same animal and were instrumental in feeding [20] and genetics studies [38] and other research on growth rates, site fidelity, and wound healing. The external tags were especially helpful in obtaining resighting information from the local ecotourism industry (2004–09), adding to the data collected by project biologists. Given the uncertainty of tag retention rates, however, our conventional tag data were deemed not suitable for deriving any meaningful estimates of population size and instead aerial surveys have been utilized for this purpose [18].

The PSAT failure rate in this study (7 out of 35 tags, 20% non-reporting overall) was less than the 36.8% failure rate reported by Wilson and his colleagues (2006) [6] for a comparable whale shark study, possibly reflecting improvements in tag technology. We began using the Mk10-PAT (Wildlife Computers) PSAT in 2006, and we experienced a lower failure rate with that model (4 out of 29 tags, 14% non-reporting). The premature detachment of half of our reporting PSATs (14 out of 28) is similar to results from other whale shark studies [36], [39] and may be due to incomplete anchoring of the tag’s dart in the subdermal tissue, biofouling that increases drag, and/or the animal actively attempting to remove the tag by rubbing it off. Delayed transmission of data from tags that popped up on time was likely the result of accumulated biofouling that affected the tag’s orientation at the surface, and/or the tag’s wet/dry sensor, and therefore its ability to transmit. This is supported by our observations in August 2009 of a PSAT, deployed 42 days earlier and still on the shark, which already had a thin layer of green algae despite the tag being coated with antifouling paint. Wilson et al. (2006) [6] similarly suggested that rapid biofouling in tropical waters may have contributed to the non-reporting rate of their PSAT tags deployed on *R. typus*. The application of antifouling coatings to PSAT tags may not solve this problem entirely but is likely to have a positive effect on results. We have yet to see the perfect antifouling material for satellite tag applications in tropical environments.

### Comparison of SPOT vs PSAT through Double-Tagging

In our double-tagging experiment, the application of first the UKFSST model and then the bathymetric correction improved the accuracy of the PSAT location estimates substantially, as shown by a reduction in the RMS error of the MPT’s latitude and longitude (0.277°; 0.289°) compared to those of the raw geolocations (1.922°; 0.301°). The RMS errors associated with the MPT in our study are lower than those reported in a comparable study of whale sharks in the Indian Ocean (RMS error = 1.84° latitude and 0.78° longitude) [40]. In the latter study, two PSATs (model PTT-100; Microwave Telemetry, Inc., Columbia, MD, USA) and one SPLASH tag (Wildlife Computers) were attached to a 4–5 m male whale shark and the raw data were refined with a similar model that incorporates SST (KF-SST; n = 37 and 23). Our study’s relatively low measure of geolocation error is in part attributable to the small number of usable track days (n = 13) for the double-tagged shark, the modest geographic movements of the shark during this time period, and the animal’s limited vertical movements due to the relatively shallow depths along the track (Fig. 7). Nevertheless, our results support the notion that the UKFSST model followed by the bathymetric correction is a robust method for refining raw, light-based positions by generating reliable and statistically sound geolocation estimates [28], [41], [42].

### Size and Sex Composition of Whale Sharks

In this study, we applied conventional visual tags to a large number of whale sharks (n = 813) in a haphazard and unbiased manner such that the size (2.5–10.0 m TL;  = 6.33 m TL) and M:F sex ratio (2.6∶1; 72% males) of tagged animals should be representative of the summer population in the study area. Shark size range off the YP is similar to that off Belize (range = 3.0–12.7 m,  = 6.3 m TL) [11] and Ningaloo Reef (range = 3.0–9.7 m,  = 6.7 m TL) [23] but without the bimodal distribution (Fig. 2) sometimes seen elsewhere, e.g. at Ningaloo Reef [23] and in India [43]. A comparable male bias has been observed off the Seychelles (82%) [44], Ningaloo Reef (83%) [23], Djibouti (85%) [44], and Belize where 86% of the sharks observed are immature males [11]. Habitat segregation by sex is common in sharks [45], [46] and may be established for social, thermal or forage-related reasons [47]. In the basking shark, another filter-feeding species, sexual segregation has not been clearly demonstrated in the northeastern Atlantic, but pregnant females of this species are virtually unobserved in this population [48]. In the present study, female *R. typus* up to 10 m TL were visually tagged but large females (>8 m) were not commonly observed off the northeastern YP.

An absence or scarcity of large females has been similarly described at other aggregation sites [11], [35]. Studies of whale shark coastal aggregations have increased sharply in recent years [36] and the size, identifiability, and distribution of this species makes remote the possibility that large females inhabit some as yet unknown coastal regions. We therefore propose that this size class remains largely in offshore waters in the western Atlantic and other ocean basins. This is supported by observations from the GOC where large, apparently pregnant females (>9 m) have been documented in offshore waters south of Banco Gorda whereas only juveniles are found in the northern and central GOC [49]. A recent study in the southwest GOC by Ketchum et al. (2012) [50] found juvenile whale shark (<9 m TL in the GOC) distribution in shallow coastal waters to be correlated with diet preference, as these areas contain dense zooplankton patches which could facilitate faster growth rates in these young sharks. In contrast the adult sharks (>9 m TL in the GOC; 84% females) preferred deeper offshore habitat and fed opportunistically on the prey available there. These findings are consistent with stable isotope analyses of muscle tissue from Indian Ocean whale sharks, suggesting that as shark size increases there is a shift in their diet to prey of a higher trophic level [51]. The use of offshore habitats by large females could also be related to different thermal requirements [52] and/or alternative migratory patterns to accommodate gestation and parturition, strategies found in other shark species [53].

### Wounds from Boat Strikes

In a study off the northeastern YP, Ramírez-Macías et al. (2012) [19] reported 25% of the whale sharks observed off Isla Holbox between 2005 and 2008 displayed evidence of collisions with boats. Their report is consistent with our observations. Wounds from boat strikes have been found in other whale shark aggregation areas including Ningaloo Reef [23], the Gulf of Tadjourna, Djibouti [54] and Belize [11]. Collisions with large vessels may be of even greater concern than small boat strikes. Some of the Caribbean’s busiest shipping lanes run near or through locations off Quintana Roo where whale sharks and giant mantas aggregate to feed [55], [56]. Stevens (2007) [57] stated that human-related mortality of whale sharks, aside from directed fishing, occurs mainly through boat strikes while Speed et al. (2008) [58] recommended that mortalities by large vessel strikes should be accounted for in management of whale sharks. Observing and quantifying large vessel strikes pose significant challenges, however, as such collisions probably occur offshore and dead sharks normally sink to the bottom.

### Resightings within the Study Area and Residence Time

Philopatric behavior in sharks can be revealed through various lines of evidence including tagging studies, genetic analyses, and indications of localized stock depletion [59]. The large body of resightings data from within our study area, via both visual tagging and photo-identification, indicates that many individual whale sharks return to the northeast Yucatan in consecutive years (at least as many as six consecutive years) to utilize the summer feeding grounds. Whale sharks have been similarly observed returning to Gladden Spit in Belize [11] and Ningaloo Reef [23] in successive years. Our estimates of average residence time in the study area from visual tagging (23.6 days) and photo-identification (32.5 days) are comparable to calculations for whale sharks at Ningaloo Reef using photo-identification and an Open Robust Design model structure (33 days) [25]. Maximum duration between first sighting and last resighting of the same animal in a given year (187 and 106 days for photo-identification and visual tagging, respectively) suggests that individual sharks may reside as long as 6 months in the Yucatan study area. Although more evidence is needed to confirm this, these results together with the sheer number of animals in the aggregation [18], [19] demonstrate the importance of the YP area to the species in the northwestern Atlantic region.

### Movements into the GOM

Our study provides evidence that when whale sharks leave the YP feeding area, a large proportion of the animals move into other portions of the GOM and utilize these areas during the months of September through January. Of the 22 PSAT tags providing long-distance data, 50% (11) showed movements into the central GOM and, to a lesser extent, to northern, western and southern GOM waters (Fig. 3). The presence of whale sharks in the GOM was first reported by Gudger (1939) [9], followed later by several accounts for the upper GOM [60]–[63]. More recently in the northern GOM, *R. typus* has been reported off south Texas in June and August [64] and off Louisiana from May to November [65]. In a report by Hoffmayer et al. (2007) [8], a whale shark feeding aggregation southwest of the Mississippi River mouth in June 2006 was associated with recently spawned eggs of the little tunny (*Euthynnus alleteratus*), the same species whose eggs are eaten by whale sharks off the YP [18]. Using spatially and temporally intensive aerial surveys in continental slope waters of the northern GOM, Burks et al. (2006) [7] reported *R. typus* throughout the year but with greatest abundance during summer months. The largest aggregation (23 whale sharks) documented in that study was located about 33 km west of the Flower Garden Banks. In our study, this same area was used heavily by Sharks 9 and 10 from October to December, indicating this part of the northern GOM may contain important habitat for the species. The continental shelf waters of the northern GOM are physically and biologically dominated by the nitrate-rich input of the Mississippi River, one of the world’s largest rivers [66]. The upper GOM is also known for cyclonic and anticyclonic eddies that can generate nutrient-rich upwellings leading to localized areas of increased primary production. These productivity hotspots can be on a micro- to meso-spatial scale and contain high concentrations of zooplankton, micronekton, and higher-trophic level organisms in otherwise blue water areas [67], [68]. Although direct evidence is lacking, it is likely the presence of whale sharks in the northern GOM is related to these localized productivity events. Basking sharks have been shown to move non-randomly over long distances towards plankton prey fields of higher density through a complex series of behavioral patterns [69]. Studies have demonstrated that basking shark foraging is focused mainly in productive continental shelf and shelf-edge habitats that contain seasonal increases in zooplankton abundance [48]. The specific mechanism by which basking sharks or whale sharks are able to detect and navigate to such hotspots is unknown and is a subject requiring further research.

Our study demonstrated that some of the whale sharks utilizing the YP feeding area migrate through the GOM waters over the West Florida Shelf. Isolated sightings of *R. typus* typically >30 km off the west-central Florida coast have been reported by the public to Mote Marine Laboratory (R. Hueter, unpublished data). In early summer 2010, a pulse of sightings of whale sharks and other pelagic species occurred in this area, some only a few kilometers from shore. These rare inshore sightings off the Florida GOM coast coincided with the Deepwater Horizon oil blowout offshore, which spread 800 million liters of oil over large portions of the northeastern GOM that spring and summer. Whether these two phenomena were linked is subject to speculation, for we cannot rule out a localized burst of productivity such as generated by an upwelling event. The filter-feeding behavior of whale sharks, however, makes them highly vulnerable to oil spills and application of chemical dispersants [70], possibly resulting in avoidance of contaminated areas and alterations of the sharks’ migratory paths.

### Movements into Waters around Cuba

After moving out of the study area, at least three of the PSAT-tagged whale sharks migrated east and spent parts of October and November off the northwest coast of Cuba (Fig. 4A), which appears to be a core-use area, especially for the larger females (Fig. 6A–E). The earliest accounts of *R. typus* in Cuban waters described three specimens captured near Havana Harbor [71], [72]. Feeding whale sharks also have been associated with schools of “bonito and sardines” in three Cuban localities on the northwest coast (off Havana), the northeast coast (off Gibara and Vita), and the southeast coast (off Manzanillo) [73]. More recently, sightings of whale sharks have been reported by tuna fishermen off Cuba’s northwest coast, primarily near the shelf edge during October and November (G. González-Sansón pers. comm.) and along the south coast in the Jardines de la Reina Archipelago between October and December [74].

### Movements into the Caribbean Sea

Photo-identification data from our study showed a connection between whale sharks present in summer months off the YP and sharks observed during winter and spring off Belize (Gladden Spit) and Honduras (Utila Bay Islands), two areas with an active ecotourism trade for whale sharks and research programs that utilize photo-identification [1], [25]. The connection among these sites is supported by visual tag resightings from this study, resightings of animals tagged in Honduras by other research programs, and tagging results described from Belize [11]. Predictable aggregations of whale sharks have been documented along the Belize Barrier Reef during the months of April and May [10], [75] and off the north shore of Utila, Honduras primarily between the months of February and May [74].

Satellite tracking data for Sharks 5, 17, 21 and 35 revealed movements into other parts of the Caribbean Sea and the sharks’ use of this tropical environment for up to several months (Fig. 4B). Accounts of whale sharks off Trinidad [76], Haiti [9], and the Bahamas [77], [78] are mentioned in the early literature but substantive, contemporary reports of *R. typus* in the eastern Caribbean Sea are lacking. However, the ECOCEAN database reports encounters from several islands in this area including Aruba, Dominica, Grenada, Puerto Rico, and the US Virgin Islands. Sharks 21 and 35 showed movement into the southern Caribbean Sea suggesting a possible connection to whale shark populations observed off central and South America. Compagno (2001) [1] reported whale sharks off central Brazil, Colombia, Panama, and Venezuela. In a compilation of whale shark sightings over a 51-year period, Romero et al. (2000) [79] reported 20 specimens of *R. typus* off Venezuela between the months of August and February with most sightings from a region of highly productive upwelled water.

### Movements into the Atlantic Ocean

According to its MPT, Shark 15 (Rio Lady) traveled 7,772 km through the Caribbean Sea and into the mid-Atlantic Ocean south of the equator in 150 days, a migration requiring an estimated mean speed of no less than 51.81 km day^−1^ (minimum = 48.1 km day^−1^ based on the straight-line tagging-to-pop-up distance), one of the fastest whale shark migrations recorded to date. Analysis of the archived depth data revealed regular deep dives (1,600 m maximum) throughout the 150-day track, eliminating the possibility that this tag was not attached to the shark for the entire duration. Sequeira et al. (2013) [36] discussed the skepticism surrounding a nearly 13,000 km track for a whale shark in the Pacific Ocean reported by Eckert and Stewart (2001) [4], which was most likely due to a floating tag that had become detached from the shark. Excluding that record, the only other confirmed track longer than Rio Lady’s to date was reported by Eckert et al. (2002) [5], an 8,025 km track in the Indo-Pacific. The end-point of Rio Lady’s track is located over the Mid-Atlantic Ridge, northwest of Ascension Island. The nearest land feature is the Saint Peter and Saint Paul Archipelago (SPSPA), a group of small rocky islands [80] 543 km northwest of this tag’s January pop-up location (Fig. 5). Sightings of whale sharks have been reported from this area [81]–[83]. From February 2000 to November 2005, Hazin et al. (2008) [80] reported 54 whale sharks observed off the SPSPA ranging 1.8–14 m TL. Although sharks were sighted nearly every month of the year, the sightings at SPSPA were most common from January to June. This region is not known for upwellings or high productivity [84] but the SPSPA area is thought to be a spawning ground for the margined flyingfish (*Cheilopogon cyanopterus*) from January to March, with larvae of this species being the most abundant plankter in adjacent waters in January and February [85]. However, Hazin et al. (2008) [80] noted no filter-feeding activities by whale sharks during any of the sightings around the SPSPA.

It seems unlikely that Rio Lady migrated this extreme distance so rapidly solely to take advantage of a localized productivity event in the middle of the Atlantic Ocean on the opposite side of the equator. No other shark tagged at the YP site made the same trek. Alternatively, it is conceivable this female estimated to be 7.5 m TL made this extensive migration for reproductive purposes. Size at first maturity for whale sharks has not been conclusively established because few reliable sources of data exist on this important life history parameter [57], [86]. Beckley et al. (1997) [87] reported eight necropsied females up to 8.7 m TL that had stranded in South Africa were all immature, but failed to report what criteria were used to assess maturity status. In a subsequent study by Wintner (2000) [88], one of these same South African specimens measuring 5.77 m TL was determined to be adolescent. For male whale sharks, we observed in the YP aggregation elongated, differentiated, “knobby” claspers that extended beyond the pelvic fins on males <8 m TL, smaller than what is generally reported in the published literature for male size at maturity (e.g. Rowat and Brooks 2012 [86]). The possibility that the Atlantic population of whale sharks matures at a smaller size than the Indo-Pacific population cannot be ruled out. In addition, our study’s length estimates were made from a moving vessel on actively swimming and bending sharks and are accurate to about ±0.5 m. Rio Lady’s total length, therefore, could have been as much as 8.0 m at the time of her first satellite tagging. If the swelling we observed in Rio Lady’s pelvic region at the time of tagging was due to early pregnancy, this migration could have been for this female to give birth to her young in the open waters of the mid-Atlantic Ocean. Mating activities might also be related to this journey. Our hypothesis of a reproductive function for Rio Lady’s long migration is consistent with other observations. The scant data on pupping grounds of *R. typus* [89]–[91] suggests the use of offshore pupping areas and isolated primary and secondary nursery areas. The limited records of free-swimming neonate whale sharks have come mostly from open ocean habitats, including several from the equatorial mid-Atlantic (Fig. 5) [92], [93]. Furthermore, of the 54 whale sharks reported near the SPSPA by Hazin and co-workers (2008) [80], three had estimated lengths of 1.8–2.0 m, a juvenile size range virtually absent from the rest of the whale shark literature [89]–[91].

In a recent review of the biogeography of the whale shark, Sequeira et al. (2013) [36] hypothesized the world’s *R. typus* comprise a single, global meta-population and suggest the SPSPA area may be an important trans-Atlantic thoroughfare for this species. If this is the case, we cannot discount the possibility that Rio Lady’s journey was part of a longer trans-oceanic passage to access mid-Atlantic and/or west African feeding grounds. The ECOCEAN photo-identification library reports mid-ocean whale shark encounters from Ascension Island and St. Helena. Off western equatorial Africa, *R. typus* has been reported in the Gulf of Guinea and off the coasts of Côte d’Ivoire, Gabon and Angola [1]. In a paper describing the sightings of 5–7 m whale sharks in a relatively localized area off Angola, Weir (2011) [94] suggested the presence of *R. typus* there might be related to high primary productivity caused by the convergence of the Angolan and Benguela currents [95] and/or freshwater outflow from the Congo River [96].

### Overall Rates of Movement

With calculations based solely on the distance between tagging and pop-up, the sharks’ minimum mean rate of travel was about 10.3 km day^−1^. Based on their MPTs, our 22 sharks moved at a mean rate of about 28.6 km day^−1^(1.19 km hr^−1^). These results are somewhat higher than whale shark movement rates estimated by Eckert and Stewart (2001; 23.8 km day^−1^) [4], Wilson et al. (2006; 26.3 km day^−1^) [6], and Brunnschweiler et al. (2009; 13.8 km day^−1^) [39], although direct comparisons among studies are complicated by differing tag types, alternative methods of calculating travel rate, and varying sample size. The average movement rate calculated from the SPOT5 track in our study (1.93 km/h) is comparable to the average rates for whale sharks reported by Rowat and Gore (2007; 0.99–2.37 km hr^−1^) [97] and Hsu et al. (2007; 1.18–1.44 km hr^−1^) [98], with both studies using similar methods (SPOT2; Wildlife Computers).

### Oceanographic Factors Associated with Whale Shark Movements

The cues that trigger whale sharks to leave the YP feeding area from August through October are unknown. Decreasing water temperature, day length, or prey density may all be factors but the sharks do not all leave during the same week or even the same month, and in fact, some sharks have been sighted in the area in early winter (de la Parra, unpublished data). When sharks do leave, however, our habitat utilization distributions indicate the primary migration corridor away from the tagging area is to the north and along the eastern edge of the Campeche Bank (Fig. 6). Other core-use areas include those off northwestern Cuba, northwest of the Campeche Bank, and in the northwestern GOM, areas which are near continental shelf edges (Fig. 6). The dynamic physical processes associated with shelf edges generate upwellings of nutrient-rich water to the surface [15], that can result in localized increases in planktonic biomass [100]–[102]. A correlation between whale shark distribution and proximity to the shelf edge has been described for the northern GOM [7], [103] and may be related to pulses of productivity associated with these areas.

In Ningaloo Reef, correlations between whale shark movements and the retreat of warm SST isotherms toward the equator have been described [6]. In the Indian Ocean, SST was similarly found to be the most suitable predictor of whale shark habitat while surface chlorophyll-*α* concentrations were less reliable [104]. Chlorophyll-*α* appears to be a poor proxy for zooplankton availability because the trophic links between the two are not necessarily direct or temporally and spatially synchronized [104], [105]. In whale sharks tracked from Ningaloo Reef, surface geostrophic currents do not appear to be used by the animals as an aid to migration [105]. At Ningaloo, the Southern Oscillation Index, effectively a measure of El Niño and La Niña climatic processes, along with wind shear have been found to have the greatest influence on whale shark abundance, by affecting along-shelf currents that re-suspend nutrients resulting in a pulse of productivity [106], [107]. Comparable studies with basking sharks have demonstrated they are not indiscriminant planktivores but selective filter-feeders that choose the richest plankton patches, which are often associated with localized thermal fronts [108]. On a broader scale, basking sharks regularly undertake extensive horizontal movements to temporally discrete hotspots of productivity located in continental shelf-edge habitats [48], [109].

### Conclusions and Recommendations for Whale Shark Conservation

The summer feeding aggregation of juvenile and adult male and female whale sharks off the YP is the largest currently known to science for this species [18], [20]. Conventional tagging and photo-identification suggest that *R. typus* can remain for as long as six months in the area, many of the animals demonstrate philopatric behavior by coming to the site for as many as six years in a row, and the sharks move back and forth from northwestern Caribbean areas such as Belize and Honduras. Satellite tracking up to 190 days after tagging has demonstrated movements away from the YP feeding site and habitat utilization throughout the GOM and Caribbean Sea into winter months, perhaps to take advantage of localized productivity hotspots. Several satellite tracks show whale sharks returning to the YP area later in the winter of the same year, a finding that is supported by sporadic on-water reports of sharks off the YP during cooler months.

The identification of essential habitat for mature female whale sharks, especially their mating and pupping grounds, is an important question for research and conservation of the species. The long-distance transequatorial migration of a possibly mature, pregnant female into the South Atlantic Ocean is hypothesized to have been for reproductive purposes. This migration demonstrates potential gene flow between distant areas and supports genetic studies showing little differentiation between distinct geographic populations [38], [110]. Basking sharks in the North Atlantic also may be making ocean basin-scale migrations across the equator to mate or give birth in as yet unknown locations in the South Atlantic Ocean [111]. The scarcity of neonate or very young whale sharks in coastal areas [86] strongly suggests that pupping areas for these sharks are located in offshore, pelagic habitats. Size at birth for *R. typus* is variable but appears to range 46–60 cm TL [112], [91], [113], a size that could make the young pups vulnerable to heavy predation in biologically rich areas. Pelagic environments with less megafauna but sufficient amounts of food for young whale sharks may serve as primary nursery areas for the species. The whale shark’s relatively high fecundity (as many as 300 young per litter) [112] could help offset higher rates of natural mortality in young stages as compared with other elasmobranch species.

The whale shark’s summer feeding grounds off the Yucatan Peninsula of Mexico, where the Gulf of Mexico meets the Caribbean Sea, is one of the most important population centers for *R. typus* in the world. Protection of this vital area may benefit the species as a whole. The broad movements of whale sharks from this region and through multiple geopolitical boundaries underscores the need for multi-jurisdictional approaches to whale shark conservation. The 2010 environmental disaster caused by the Deepwater Horizon oil spill in the northeastern GOM [70], less than 600 km from the Yucatan feeding grounds, drew attention to the potential vulnerability of whale sharks to habitat change and degradation. Continued research on the behavior and ecology of these ocean travelers provides the foundation for best management practices for their protection.
