# Correction: Horizontal Movements, Migration Patterns, and Population Structure of Whale Sharks in the Gulf of Mexico and Northwestern Caribbean Sea

**DOI:** 10.1371/annotation/491b9b6c-7f77-4fb0-b336-572078aec830

**Published:** 2013-11-07

**Authors:** Robert E. Hueter, John P. Tyminski, Rafael de la Parra

Multiple funding organizations and a grant were incorrectly omitted from the Funding Statement. The following should be read with the Funding Statement:

"The Natural Protected Areas National Commission of Mexico (SEMARNAT/CONANP) provided personnel, biological station facilities, boats, fuel and conventional tags during the early stages of the work (2003-2009). Those stages also were supported in part by a Mexico Small Grants Program funded by the UNDP/Global Environment Facility, as provided by the Mexican NGO SER de Quintana Roo A.C. The authors sincerely regret this omission."

In addition, the following Acknowledgments were incorrectly omitted from the Acknowledgments Statement:

"The authors were remiss in acknowledging the assistance and leadership of Francisco Remolina Suarez, Jaime Gonzalez Cano and Jose Juan Perez Ramirez of the Natural Protected Areas National Commission of Mexico in helping to launch and coordinate this collaborative study." 

